# Hypercalcemia in a patient with a bowing femur

**DOI:** 10.1002/ccr3.3569

**Published:** 2020-11-23

**Authors:** Maroua Slouma, Safa Rahmouni, Rim Dhahri, Maissa Abbes, Imen Gharsallah, Leila Metoui, Bassem Louzir

**Affiliations:** ^1^ Department of Internal Medicine Military Hospital Tunis Tunisia; ^2^ Tunis El Manar University Tunis Tunisia

**Keywords:** hypercalcemia, hyperparathyroidism, Paget’s disease

## Abstract

Hypercalcemia in PDB is rare; its occurrence requires thorough investigations as it may reveal several diseases, such as primary hyperparathyroidism, malignant transformation, metastases, or myeloma.

## Graphical abstract

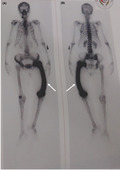

## 1 INTRODUCTION

Paget’s disease of bone (PDB) is a benign bone dystrophy. The occurrence of hypercalcemia in PDB can be alarming and needs to be investigated. We describe the case of a patient with concomitant PDB, primary hyperparathyroidism, and monoclonal gammopathy of undetermined significance, revealed by hypercalcemia.

Paget’s disease of bone (PDB) is a benign bone dystrophy that typically affects patients after 55 years.^1^ There is a striking heterogeneity in the incidence of Paget's disease in regions parts of the world. During the last years, there has been a decline in the prevalence and severity of PDB,^1^, ^2^. It can be responsible for condensing, hypertrophic, and deforming lesions. The diagnosis is based on clinical, biological, and radiological features.^2^, ^3^ PDB complications generally consist of fractures, neurological complications, and rarely, transformation into osteosarcoma. Hypercalcemia can be associated with the disease and may be caused by several conditions such as immobilization, fracture, or dehydration.^4^ However, hypercalcemia may reveal the coexistence of other metabolic bone diseases, malignant conditions such as bone metastases, or malignant transformation into osteosarcoma.

We describe here a challenging case of a patient with concomitant PDB, primary hyperparathyroidism (HPT), and monoclonal gammopathy of undetermined significance (MGUS) revealed by hypercalcemia.

## 2 CASE PRESENTATION

A 80‐year‐old woman, with no significant past medical history, presented with a two‐year history of pain in the left knee and thigh.

Physical examination revealed a painful limitation of range of motion of the left hip and knee. The body temperature was 37.1°C. The neurological examination was unremarkable. There was no lymph node enlargement nor skin abnormalities.

**Figure 1:**
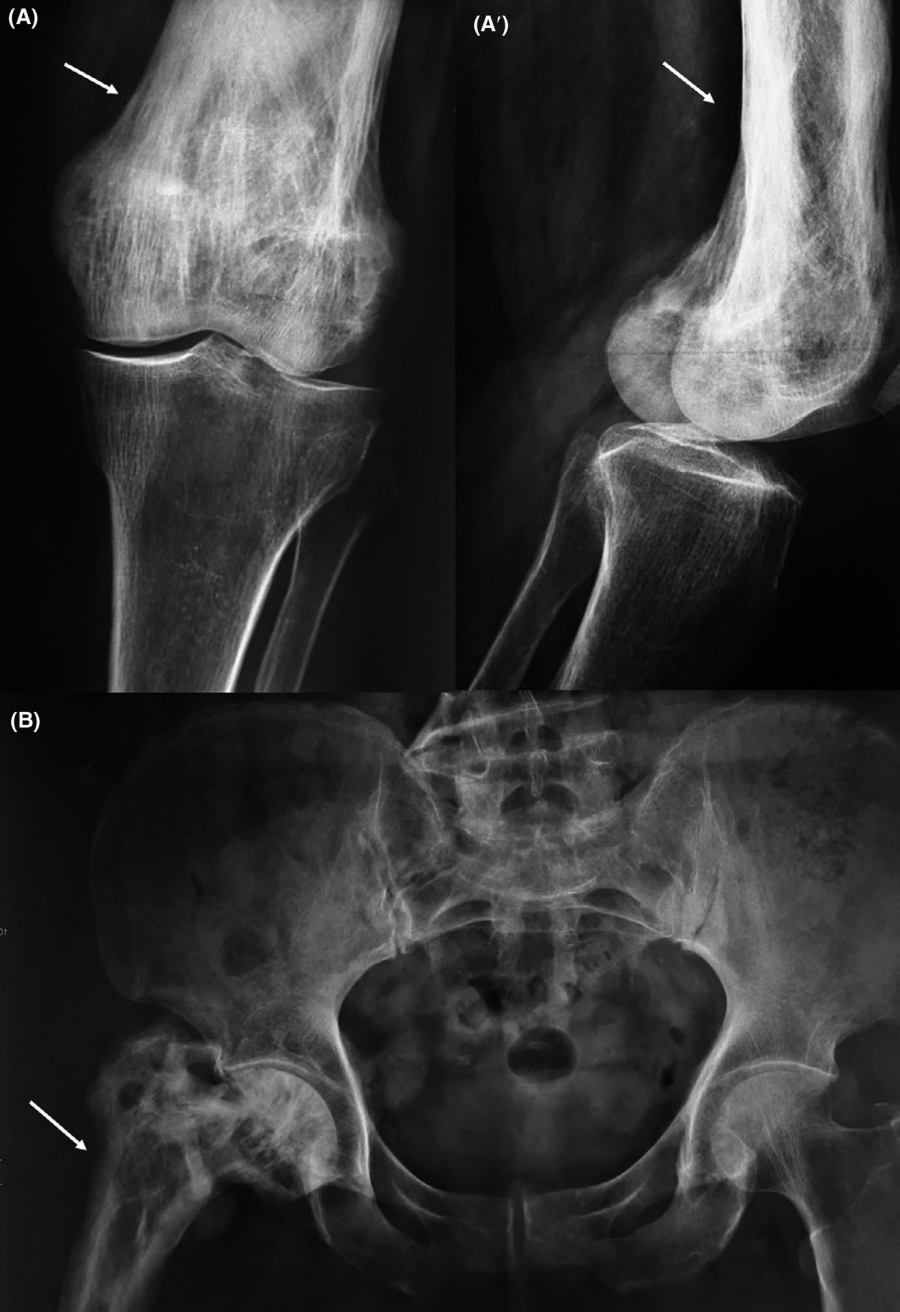
(A) Knee’s radiographs (anteroposterior view (A) and lateral view (A’) showing bony expansion with cortical thickening and loss of corticomedullary differentiation of the lower extremity of the left femur. (B) Pelvic radiograph showing a mixed appearance, including osteolytic and osteoblastic lesions, bone expansion and deformity of the left femur.

Knee’s radiographs showed bony expansion with cortical thickening and loss of corticomedullary differentiation of the lower extremity of the left femur (Figure 1A).

The pelvic X‐rays showed areas of bone demineralization and hypertrophy of the left femur (Figure 1B).

Laboratory examinations revealed hypercalcemia of 2.99 mmol/L [2.09‐2.54 mmol/L], hypophosphatemia of 0.63 mmol/L [0.8‐1.5 mmol/L], and elevated serum alkaline phosphatase (ALP) level of 284 UI/L [38‐167 U/L]. She had increased parathyroid hormone levels of 512 ng/L [N < 50 ng/L]. Erythrocyte sedimentation rate and C‐reactive protein were within the normal range. The complete blood cell count, liver, and renal tests were unremarkable.

**Figure 2:**
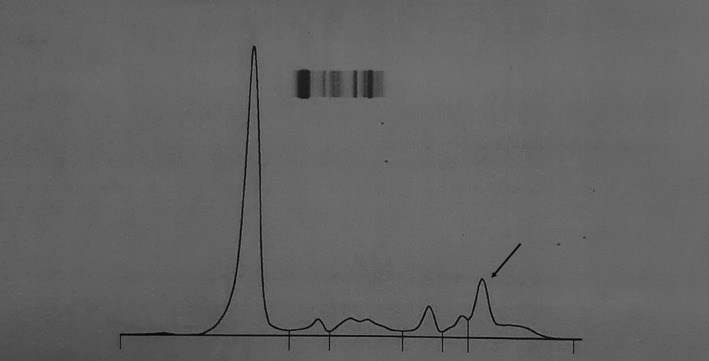
Protein electrophoresis showing a narrow peak in the gamma globulin region (black arrow)

The serum protein electrophoresis revealed a single narrow peak in the gamma globulin region. The serum gamma globulin levels were 10.45 g/L (Figure2). Immunoelectrophoresis demonstrated immunoglobulin G monoclonal protein. The bone marrow aspirate showed 6% of plasma cells.

**Figure 3:**
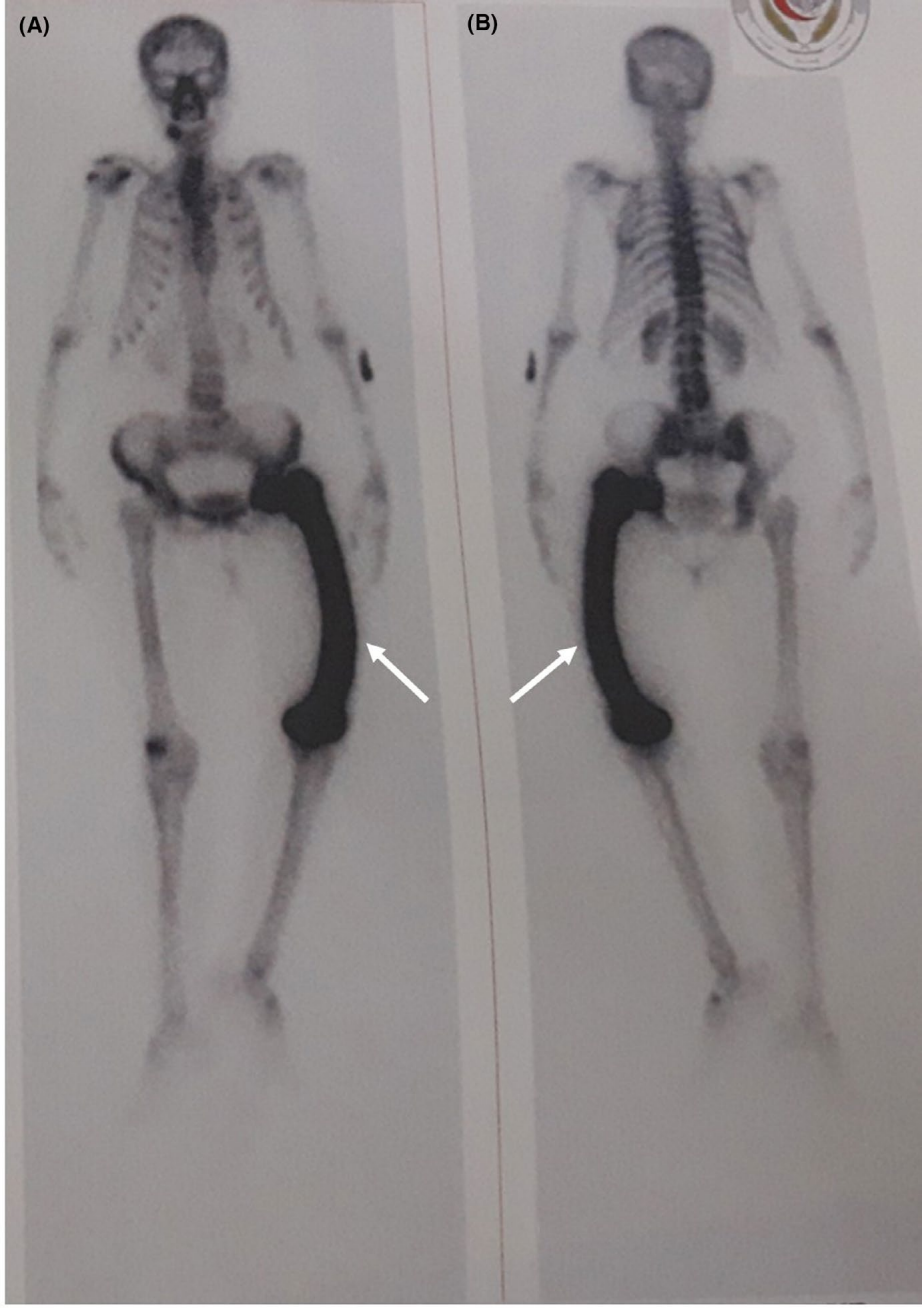
Radionuclide bone scintigraphy revealing diffusely increased homogeneous uptake (arrow) in the left femur with bowing deformity (A: anterior view; B: posterior view)

The radionuclide bone scintigraphy revealed diffusely increased homogeneous uptake in the left femur with bowing deformity (Figure 3). Magnetic resonance imaging (MRI) of the spine did not demonstrate any abnormalities suggestive of multiple myeloma (MM). A cervical ultrasound was performed showing parathyroid adenoma. Thus, the diagnosis of monostotic Paget’s disease of bone associated with hyperparathyroidism and monoclonal gammopathy of undetermined significance was established. The patient received a single intravenous infusion of Zoledronic acid with normalization of serum calcium level of 2.09 mmol/L.

Three months later, the patient reported a significant improvement in bone pain. The serum alkaline phosphate and calcium levels were within the normal range. We did not observe any additional complications. Then, the patient was lost to follow‐up.

## 3 DISCUSSION

Paget’s disease of bone (PDB) is benign bone dystrophy. Its prevalence increases with age and is around 0.3% after 55 years.^1^ Deformity, hypertrophy, and condensation of the bone are the hallmarks of this disease. While the etiopathogenesis is not fully understood, both genetic and environmental factors seem to play an important role in this bone disorder.^2^, ^3^, ^4^

The diagnosis is usually made incidentally since the disease remains asymptomatic for a long time. Clinical presentation is various and not specific. It may include bone pain, limbs or skull deformities, and fractures. Neurological symptoms can occur as a result of mechanical compression of neural tissues or due to ischemia. An isolated elevation of total alkaline phosphatase may also reveal the disease.^4^ The axial skeleton is the most involved site.^5^

Radiological features commonly include y bone hypertrophy, cortical thickening, and loss of corticomedullary differentiation. Bowing deformities occur frequently in the long bones of the extremities and can lead to osteoarthritis and pain.^2^ Radiographic findings PDB is characterized by three distinctive phases. The first phase is marked by an increased osteoclastic activity leading to geographic osteolysis. The intermediate mixed phase is characterized by a lytic‐sclerotic appearance, cortical thickening, hypertrophy, and loss of corticomedullary differentiation. The third phase has pronounced cortical and medullary sclerosis with significant bone expansion.^5^

The diagnosis of PDB is based on clinical and radiographic features.^2^, ^3^ The disease can be monostotic or polyostotic. Radionuclide bone scintigraphy, performed to assess the extent of the disease, shows a nonspecific increased radioisotope uptake.^4^

The main differential diagnosis of PDB is condensing bone metastases, notably in the presence of high serum calcium levels.^6^ Hypercalcemia is not a frequent feature of PDB. When it occurs, it should raise the possibility of an association with another condition such as bone metastases, primary hyperparathyroidism, and multiple myeloma. The diagnosis may be challenging since the association between PDB and bone metastases is not uncommon in elderly patients. The primary sites of malignancy in patients with PDB are the breast,^7^ prostate,^8^ lung,^9^ and pancreas.^10^

In addition, hypercalcemia can be due to neoplastic transformation, especially in patients with long‐standing active disease. Indeed, malignant degeneration of PDB into osteosarcomas, or less frequently, fibrosarcoma or chondrosarcoma, has been described in 1% of cases.^11^, ^12^ It usually affects the femur, tibia, humerus, and skull. Several signs and symptoms should suspect this complication, such as fever, exacerbation of pain, increased calcium levels, and inflammatory biomarkers.^13^, ^14^ Radiographic changes, notably the appearance of new osteolytic lesions with periosteal reaction, support the diagnosis of malignant transformation of PDB.^12^

The association between PDB and MM is scarce and has been reported in a few case reports.^15^, ^16^, ^17^, ^18^ Although these two diseases may share overlapping clinical signs, their radiological features are quite different. While both diseases are characterized by an increased osteoclastic activity, the bone resorption in PBD is followed by defective bone repair. Thus, the new bone is disorganized, thicker but weaker.^16^

Whereas MM is typically characterized by lytic bone lesions (skull, ilium, ribs, vertebrae).^15^

In our case, the diagnosis of hypercalcemia related to MM was initially suspected, given the existence of a narrow peak on protein electrophoresis in the gamma globulin region. However, the bone marrow cytology, MRI findings, and laboratory examinations were consistent with the diagnosis of MGUS. To our knowledge, this association has not been reported previously.

Apart from malignant conditions, hypercalcemia in patients with PDB may be due to the coexistence of metabolic bone disease, such as primary hyperparathyroidism, as in our case.^19^ This association has been described since 1934 and occurs in 2.2%‐6% of patients with PDB.^20^ It can be caused by parathyroid adenoma or diffuse hyperplasia of the parathyroid.^20^ These two entities share similar clinical presentations, such as bone pain and fractures.^21^ They also share histological features, including predominant bone marrow fibrosis and increased vascularity.^21^ The cardinal biochemical features of this disease are increased levels of serum calcium, depression of serum phosphate, excessive urinary excretion of calcium and phosphorus, along with high levels of parathyroid hormone.

The diagnosis is mainly based on biological findings.^21^

Osteitis fibrosa cystica is the classic bone complication of long‐term primary HPT occurring in 1.5% of cases.^21^ Other radiographic changes include salt‐and‐pepper appearance of the skull, subperiosteal resorption of the phalanges, osteolysis of the distal clavicles, and cysts.^22^, ^23^ Diffuse demineralization and pathological fractures may also be seen.^24^

In comparison with PDB, imaging features of HPT do not typically show sclerosis or hypertrophy. However, HPT can be responsible for deformity.^25^

Both Radionuclide bone scans can show homogeneous uptake in both diseases.^26^

With successful treatment of PDB, bone scintigraphy should show a reduction in the radionuclide uptake.^27^

Rarely, hypercalcemia may occur in patients with active polyostotic PDB during immobilization, fracture, or dehydration.^4^, ^5^, ^6^, ^7^, ^8^, ^9^, ^10^

Regarding the therapeutic management of PDB, bisphosphonates are indicated in the presence of pain and increased ALP.^2^, ^3^ Reid et al showed that a single infusion of zoledronic acid leads in most cases to rapid and sustained clinical and biological remission.^28^

Furthermore, bisphosphonates are effective agents for the treatment of hypercalcemia related to HPT. Studies have reported a significant decrease in both calcium and ALP levels during the first three months of alendronate treatment.^29^

## 4 Conclusion

The association of PDB, primary HPT, and benign monoclonal gammopathy, as observed in this case, was not previously reported.

We highlight here the possible co‐occurrence of multiple metabolic bone diseases in the same patient. The diagnosis is challenging as these diseases may share overlapping symptoms. Although hypercalcemia may occur in PDB, several conditions should be considered, such as coexistent metastases, malignant transformation, or primary HPT.

## CONFLICT OF INTEREST

None.

## AUTHORS' CONTRIBUTIONS

Safa RAHMOUNI has drafted the work. ‐ Maroua SLOUMA and Maissa ABBES have substantively revised the work. ‐ Rim DHAHRI has made substantial contributions to the analysis of data. ‐ Imen GHARSALLAH has made substantial contributions to the acquisition of data. ‐ Leila METOUI has made substantial contributions to the design of the work. ‐ Bassem LOUZIR has made substantial contributions to the conception of the work.

## ETHICAL APPROVAL

Ethical approval was obtained from the Scientific and Ethical Committees of the hospital.

## Data Availability

The datasets used and/or analyzed are available from the corresponding author on reasonable request.
